# Comparison of rapid immunodiagnosis assay kit with molecular and immunopathological approaches for diagnosis of rabies in cattle

**DOI:** 10.14202/vetworld.2016.107-112

**Published:** 2016-01-31

**Authors:** Ajaz Ahmad, C. K. Singh

**Affiliations:** Department of Veterinary Pathology, College of Veterinary Science, Guru Angad Dev Veterinary and Animal Sciences University, Ludhiana - 141 004, Punjab, India

**Keywords:** cattle, diagnosis, fluorescent antibody technique, heminested reverse transcriptase, immunohistochemistry, rabies

## Abstract

**Aim:**

Presently, diagnosis of rabies is primarily based on, conventional fluorescent antibody technique (FAT), immunopathological and molecular techniques. Recently, rapid immunodiagnostic assay (RIDA) - A monoclonal antibody-based technique has been introduced for rapid diagnosis of rabies. The present investigation is envisaged to study the efficacy of RIDA kit for the diagnosis of rabies in cattle.

**Materials and Methods:**

About 11 brain samples from cattle, clinically suspected for rabies, were screened by the FAT, Heminested reverse transcriptase polymerase chain reaction (HnRT-PCR), Immunohistochemistry (IHC), and RIDA.

**Results:**

The sensitivity for detection of rabies from brain tissue by RIDA was 85.7% as compared to 100% by IHC as well as HnRT-PCR. The accuracy of detection of rabies by RIDA was 91.6% as compared to 100% that of IHC and HnRT-PCR, whereas specificity of RIDA was 100% like that of the IHC and HnRT-PCR.

**Conclusion:**

Despite a comparatively low-sensitivity and accuracy of RIDA, latter can still be useful in screening a large number of field samples promptly. However, it is recommended that negative results with RIDA in cattle need to be authenticated by suitable alternative diagnostic approaches.

## Introduction

Rabies is the most important zoonotic disease of animals that has a significant impact on human beings. Authentic diagnostic approaches, therefore, have to be employed to distinguish this disease from other encephalitic conditions.

A rapid immunodiagnostic assay (RIDA) using a specific monoclonal antibody against rabies virus (RABV) is commercially available (Bio note, Korea). RIDA kit is rapid and simple and does not require any special equipment or technical expertise [1]. The efficacy of the diagnostic kits might vary with different species.

Immunohistochemistry (IHC) is a sensitive diagnostic technique which can demonstrate rabies antigen in fixed paraffin embedded tissue sections. In cattle, the distribution of viral antigen was revealed either in granular form or as inclusion bodies in the cerebellum, brain stem, hippocampus, and cerebrum [2-4].

Heminested reverse transcriptase polymerase chain reaction (HnRT-PCR) has been used to diagnose RABV worldwide due to its sensitivity and immense versatility and can even be useful for examining paraffin-fixed archived and decomposed samples [5]. The nucleoprotein (N) gene of RABV is targeted for diagnosing and analyzing hereditary characteristics and antigenic properties since this gene is highly conserved and combined with encapsidation of genomic RNA [6,7]. Recently, RIDA - A monoclonal antibody-based technique has been introduced for rapid diagnosis of rabies. The efficacy of the assay might vary in different species.

The present study was, therefore, envisaged to study the efficacy of RIDA in cattle where in comparison of its sensitivity, specificity and accuracy of detection of RABV in clinically suspected cattle with molecular technique *viz*. HnRT-PCR and an immunopathological technique *viz*. IHC.

## Materials and Methods

### Ethical approval

The study was approved by Institutional Animal Ethics Committee under memo no. IAEC/2014/241-70, Dated 04/12/14.

### Sample collection

Brain tissue samples of cattle (n=11) suspected for rabies were collected from post-mortem hall, Department of Veterinary Pathology, Guru Angad Dev Veterinary and Animal Sciences University (GADVASU), Ludhiana, Punjab and different dairy farms of Punjab, India, between January 2014 and January 2015. All brain samples were stored at −20°C in the laboratory for further processing. Diagnostic tests like fluorescent antibody technique (FAT), HnRT-PCR and RIDA were carried out to detect the RABV infection. Direct FAT is a gold standard [8], thus, the sensitivity of RIDA kit was analyzed in comparison to FAT in brain tissue samples. For histopathology and IHC, brain tissue samples were fixed and stored in 10% neutral buffered formalin for further analysis.

### Fluorescent antibody test

FAT was carried out on fresh brain sample following the standard protocol [9]. Briefly, glass slides with impression smears of brain tissue were placed in coplin jar containing acetone and fixed at −4°C for 1 h. Positive slides from a known rabies positive case and negative slide from a normal and uninfected animal were used as a positive and negative control, respectively. The slides were air-dried, incubated with lyophilized anti-rabies nucleocapsid conjugate (Bio-Rad, France) for 35 min at 37°C in a humid chamber and washed with phosphate buffered saline (PBS) in three successive washes for 5-10 min. The slides were rinsed with distilled water, air-dried and a cover slip was mounted by adding buffered glycerol on the smear. The slides were visualized under an immunofluorescent microscope (Zeiss) for bright apple-green, round to oval bodies. Positive and negative controls were run together with the test specimens.

### Histopathology

Brian tissues samples including cerebellum, cerebrum, and hippocampus were collected and fixed in 10% neutral buffered formalin solution and given overnight washings under tap water. Dehydration of samples was done through ascending grades of alcohol (70%, 80%, 90%, and absolute alcohol) followed by clearing with acetone. Tissues were embedded in paraffin wax (Leica Microsystem) for further processing. Approximately, 4-5 µ thick sections were cut, stained with routine hematoxylin and eosin (H and E) staining technique [10] and examined by BX61 Research Photomicrograph Microscope System (Olympus Corporation, USA), the facility provided by the department.

### IHC

Anti-rabbit polyclonal antisera raised in Rabies Research-cum-Diagnostic Laboratory of the Department of Veterinary Pathology, GADVASU, Ludhiana was used as primary antibody in IHC. Advanced SS^™^ One step polymer horseradish peroxidase (HRPO) IHC detection system (BioGenex Laboratories Inc., San Ramon, California, USA) counterstained with Gill’s hematoxylin was used. IHC was done as recommended by the manufacturer with minor modifications. Formalin-fixed brain samples were thoroughly washed in running water; dehydrated in ascending grades of alcohol and acetone; cleared in benzene and embedded in paraffin at 58°C [11,12]. Paraffin-embedded tissues were cut into 5 µm thick sections, and sister sections were taken on Superfrost/Plus, positively charged, microscopic slides (Fisher Scientific, USA). The sections were deparaffinized and rehydrated by immersing in 250 ml EZ-AR common solution at 70°C for 10 min in EZ-Retriever R System V.2.1. Subsequent antigen retrieval was done in citrate buffer (0.01 M, pH 6.0-6.2) at 95°C for 10 min and at 98°C for 5 min in EZ-Retriever R System V.2.1. Three washing were given in PBS buffer for 3 min each. The endogenous tissue peroxidases were inactivated by immersing slides in 3% hydrogen peroxide solution in methanol for 15 min at room temperature in humid chamber followed by three washings with PBS buffer for 3 min each. Non-specific binding was blocked by incubating sections with ready-to-use power block for 10 min at room temperature in the moist chamber. On one section of each slide, primary polyclonal rabbit anti-rabies antibody of 1:1000 dilution (in PBS) was added, and slide was incubated for 1 h in moist chamber at room temperature, whereas, on the other section of each slide, PBS without primary antibody was added, so as to serve as a negative control. The sections were washed thrice in PBS buffer for 3 min each, and thereafter, incubated with polymer HRP (Super Sensitive label, One Step Polymer-HRPO Reagent) for 30 min at room temperature in the moist chamber, followed by three washing in PBS buffer for 3 min each. The antigen-antibody peroxidase reaction was developed with a freshly prepared 3,3-diaminobenzidine (DAB) solution by mixing two drops of DAB chromogen with 1 ml of DAB buffer supplied by the manufacturer adding 5 ml hydrogen peroxide. Sections were washed in distilled water for 5 min and counterstained with Gill’s hematoxylin (Merck, Germany) for 30 s and washed in running tap water for 5 min. Finally, the slides were dehydrated in ascending grades of alcohol, cleared in xylene, mounted in DPX and examined under an advanced microscope (BX 61, Olympus Corporation, USA).

### Extraction of viral RNA

Total RNA was extracted from brain tissue using Trizol reagent (Invitrogen, USA) following manufacturer’s instructions with minor modifications. Briefly, 0.1 g brain tissue was homogenized with 1 ml Trizol and 200 µl chloroform (Ambion Life Technologies, USA) was added. After centrifugation of the sample at 10,000 rpm for 15 min, the top aqueous layer was recovered, and RNA was precipitated by adding 0.5 ml isopropanol. The sample was spun at 10,000 rpm for 10 min, the liquid removed and the pellet washed with 1 ml of 75% ethanol. The dried RNA pellet was dissolved in 50 µl sterile RNase free water. RNA concentration was measured using Nano Drop Spectrophotometer (Nano Drop Technologies, CA) in ng/µl. The quality of RNA was checked as a ratio of OD 260/280 and stored at −80°C. RNA was converted into cDNA using High-Capacity cDNA Reverse Transcription Kit with RNAse inhibitor (Applied Biosystems, USA).

### RNA Amplification

Amplification of 2 µl of reverse-transcribed cDNA template was performed in a final volume of 25 µl; 12.5 µl ×2 PCR mix (GoTaq Green Master Mix, Promega), 1.0 µl of each forward and reverse primer (JW12 and JW6) with 10 pmol concentration, and nuclease free water was added to make final volume of 25 µl. The amplification was performed in a thermal cycler with cycling conditions of initial denaturation at 94°C for 3 min; 35 cycles denaturation at 94°C for 30 seconds; annealing at 56°C for 45 s and elongation at 72°C for 20 s. Final elongation was performed at 72°C for 3 min. For HnRT-PCR, similar quantities of the PCR mixture constituents except 2 µl of the primary PCR product as template and JW12 as forward and JW10 as reverse primer were used (Table-1). Thermocyclic conditions were kept same as that of primary PCR. PCR amplified products were visualized in 1% agarose gel electrophoresis after ethidium bromide staining of 586 bp amplicons.

**Table-1:** Primers used for HnRT-PCR to target N gene.

Primer	Nucleotide sequences (5’ -3’)	Nucleotide position	Sense	Size of amplicon (bp)
JW 12	5’ATGTAACACCCCTACAATG3’	55-73	+	586
JW 6	5’CAATTGGCACACATTTTGTG3’	660-641	−	
JW 10	5’GTCATCAGAGTATGGTGTTC3’	636-617	−	

HnRT-PCR=Hemi-nested reverse transcriptase polymerase chain reaction

### RIDA kit

A commercial RIDA kit was used the following the manufacturer’s direction (Bionote, Korea). Briefly, a swab supplied with the kit was dipped into 10% homogenate of brain samples. The content of swab was shifted to an enclosed proprietary buffer of RIDA kit. A 100 µl aliquot of the sample was transferred to the sample well. The appearance of two lines, 5 min after addition of the brain samples, was considered positive result whereas the formation of one line was considered as a negative result [13].

### Calculation of sensitivity, specificity, and accuracy

Sensitivity was calculated as [TP/(TP+FN)] × 100. Specificity was calculated as [TN/(TN+FP)] × 100. Accuracy was calculated as [TP+TN/(TP+FP+FN+TN)] × 100 wherein TP was true-positives; FN was false-negatives; TN was true negatives and FP was false positives as determined by the reference assay, i.e., FAT.

## Results

Out of 11 cattle brain samples screened by FAT, only six cows (54.54%) (07RL14, 15RL14, 19RL14, 21RL14, 42RL14 and 02RL15) were confirmed to be rabid (or TP). Same six samples were detected as positive by IHC as well as by HnRT-PCR. However, RIDA could detect rabies only in five samples. Further, the intensity of the test lines, in positive samples, also varied in different field samples. The sensitivity of detection of rabies by IHC, HnRT-PCR, and RIDA was 100%, 100% and 85.71% respectively (Table-2). Specificity of detection of rabies by IHC, HnRT-PCR, and RIDA was 100% in all the tests. The accuracy of detection of rabies by IHC and HnRT-PCR was 100% and by RIDA, was 91.66% (Tables-3-5).

**Table-2:** Test results of IHC, HnRT-PCR and RIDA in comparison with FAT.

FAT	IHC		HnRT-PCR		RIDA		Total
		
	P	N	P	N	P	N	
P	06	00	06	00	05	01	06 [TP]
N	00	05	00	05	00	05	05 [TN]
Total	06	05	06	05	05	06	11

IHC=Immunohistochemistry, HnRT-PCR=Heminested polymerase chain reaction, RIDA=Rapid immunodiagnostic assay, FAT=Fluorescent antibody test, P=Positive, N=Negative, TP=True positive, TN=True negative

**Table-3:** Test results of RIDA.

Factors	Formula	Calculation	Result (%)
Sensitivity	TP/(TP+FN)]×100	5/5+1=5/6	85.71
Specificity	[TN/(TN+FP)]×100	5/5+0=5/5	100
Accuracy	TP+TN/(TP+FP+FN+TN)]×100	6+5/6+0+1+5	91.66

TP=True positive, TN=True negative, FP=False positive, FN=False negative, RIDA=Rapid immunodiagnostic assay

**Table-4:** Test results of HnRT-PCR.

Factors	Formula	Calculation	Result (%)
Sensitivity	TP/(TP+FN)]×100	6/6+0=6/6	100
Specificity	[TN/(TN+FP)]×100	5/5+0=5/5	100
Accuracy	TP+TN/(TP+FP+FN+TN)]×100	6+5/6+0+0+5=11/11	100

TP=True positive, TN=True negative, FP=False positive, FN=False negative, HnRT-PCR=Heminested polymerase chain reaction

**Table-5:** Test results of IHC.

Factors	Formula	Calculation	Result (%)
Sensitivity	TP/(TP+FN)]×100	6/6+0=6/6	100
Specificity	[TN/(TN+FP)]×100	5/5+0=5/5	100
Accuracy	TP+TN/(TP+FP+FN+TN)]×100	6+5/6+0+0+5=11/11	100

TP=True positive, TN=True negative, FP=False positive, FN=False negative, IHC=Immunohistochemistry

As such, sensitivity for detection of rabies from brain tissue by RIDA was comparatively lower (85.7%) as compared to IHC as well as HnRT-PCR (100%). Likewise, accuracy for detection of rabies by RIDA was also lesser (91.6%) as compared to IHC and HnRT-PCR (100%), whereas specificity of RIDA (100%) was comparable to IHC and HnRT-PCR (100%) (Table-6).

**Table-6:** Comparison of sensitivity, specificity and accuracy of three diagnostic techniques.

Factors	IHC (%)	HnRT-PCR (%)	RIDA (%)
Sensitivity	100.00	100.00	85.71
Specificity	100.00	100.00	100.00
Accuracy	100.00	100.00	91.66

IHC=Immunohistochemistry, HnRT-PCR=Heminested polymerase chain reaction, RIDA=Rapid immunodiagnostic assay

## Discussion

### FAT

Direct FAT is gold-standard for rabies diagnosis as recommended by the World Health Organization and Office International des Epizootics [14]. Bright apple-green, round to oval intracellular fluorescent bodies were observed in all the positive brain samples as observed earlier [15].

### Histopathology

**Figure-1:**
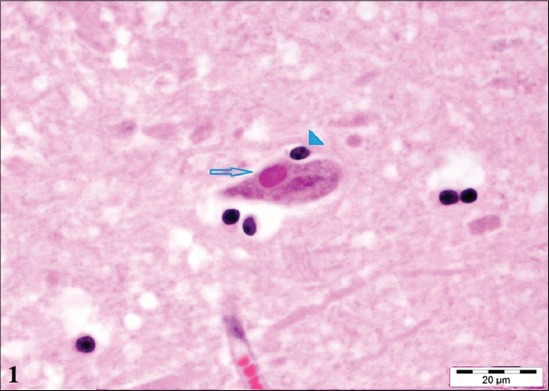
Section of the hippocampus of rabid cattle showing sharply Negri body and neuronophagia (×100).

Neuronal necrosis, Negri bodies (Figure-1), satellitosis, gliosis, neuronophagia, congestion, hemorrhage, and perivascular cuffing observed in brain samples was in accordance with the findings of Singh [16] and Sumedha [17].

### IHC

**Figure-2:**
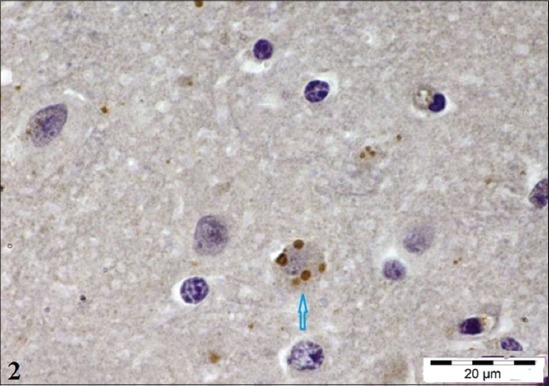
Section of the cerebellum of rabid cattle showing brown colored Negri bodies in the purkinje cells (×100).

IHC in formalin fixed paraffin embedded tissue sections has been reported to be a sensitive technique for detection of rabies antigen [18] and has been found to be of immense value for retrospective studies [19,20]. With IHC viral antigens were observed as fine granules in the cytoplasm of the neurons (Figure-2), which were not clearly visible with H and E staining.

### HnRT-PCR

**Figure-3:**
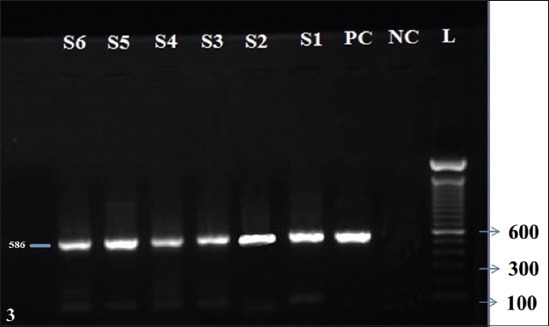
Agarose gel (1%) stained with ethidium bromide. Lane L is the 100 bp ladder, NC is the negative control, PC is a positive control, S1-S6 are the samples that are positive for fluorescent antibody technique.

In the present study, 100% agreement was observed between FAT and HnRT-PCR targeting N gene of virus (Figure-3). HnRT-PCR using a primer set that amplified the N gene of RABV was able to detect the isolates from six cows confirmed to be rabid by FAT. These isolates were detected only after both primary as well as secondary PCRs were accomplished in the assay of HnRT-PCR as reported earlier [21,22].

### RIDA

**Figure-4:**
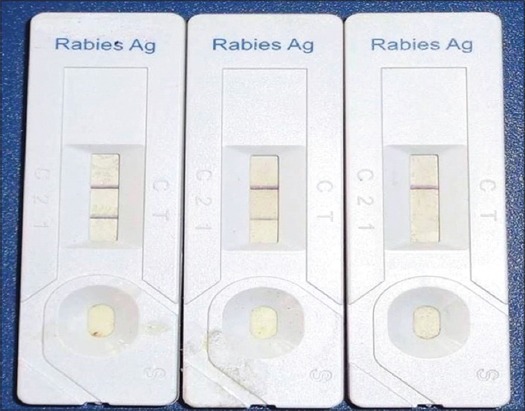
Rapid immunodiagnostic assay A and B are positive and C is negative.

The present study evaluated the efficacy of RIDA for diagnosis of rabies for use in field condition. RIDA detected rabies (Figure-4) in cattle brain samples with a sensitivity of 85.71%. The result obtained in the present study is comparable to earlier usage of RIDA kit on European mammals wherein the sensitivity of 88% reported [23]. Nevertheless, higher sensitivity of 91% was also reported, in another study, wherein, 54 brain samples were tested [24]. Despite a comparatively low sensitivity and accuracy of RIDA, it is still of use to screen a large number of field samples promptly. It is, however, recommended that any negative result with RIDA should be ruled out by confirmation with a suitable alternative diagnostic approach.

## Conclusion

It is possible to use RIDA for prompt diagnosis of rabies in the field conditions. However, the samples found negative by RIDA need to be investigated further by immunofluorescent/molecular approach for authentication/confirmation of the diagnosis of rabies.

## Authors’ Contributions

CKS designed the experiment, organized sample collection. The experiment was performed by AA under the supervision of CKS. AA collected samples and performed the diagnostic techniques. Both authors read and approved the final manuscript.
